# Experimental Warming Reduces Survival, Cold Tolerance, and Gut Prokaryotic Diversity of the Eastern Subterranean Termite, *Reticulitermes flavipes* (Kollar)

**DOI:** 10.3389/fmicb.2021.632715

**Published:** 2021-05-17

**Authors:** Rachel A. Arango, Sean D. Schoville, Cameron R. Currie, Camila Carlos-Shanley

**Affiliations:** ^1^USDA Forest Service, Forest Products Laboratory, Madison, WI, United States; ^2^Department of Entomology, University of Wisconsin-Madison, Madison, WI, United States; ^3^Department of Bacteriology, University of Wisconsin-Madison, Madison, WI, United States; ^4^Department of Biology, Texas State University, San Marcos, TX, United States

**Keywords:** thermal stress, insect microbial symbiosis, temperature tolerance, insect microbiome, climate change

## Abstract

Understanding the effects of environmental disturbances on insects is crucial in predicting the impact of climate change on their distribution, abundance, and ecology. As microbial symbionts are known to play an integral role in a diversity of functions within the insect host, research examining how organisms adapt to environmental fluctuations should include their associated microbiota. In this study, subterranean termites [*Reticulitermes flavipes* (Kollar)] were exposed to three different temperature treatments characterized as low (15°C), medium (27°C), and high (35°C). Results suggested that pre-exposure to cold allowed termites to stay active longer in decreasing temperatures but caused termites to freeze at higher temperatures. High temperature exposure had the most deleterious effects on termites with a significant reduction in termite survival as well as reduced ability to withstand cold stress. The microbial community of high temperature exposed termites also showed a reduction in bacterial richness and decreased relative abundance of Spirochaetes, Elusimicrobia, and methanogenic Euryarchaeota. Our results indicate a potential link between gut bacterial symbionts and termite’s physiological response to environmental changes and highlight the need to consider microbial symbionts in studies relating to insect thermosensitivity.

## Introduction

Temperature is a major factor influencing the distribution of many, if not all, living organisms (Doucet et al., 2009). Ecological studies characterizing species distribution and dispersal patterns rely heavily on external climactic variables which can be used to prepare risk assessments for invasive species or to identify geographic range limits for beneficial organisms. Current predictions of global climate change suggest increases in the frequency and magnitude of temperature fluctuations (Deser et al., 2012) which will directly impact seasonality, distribution, survival, and overall fitness of both beneficial and pestiferous insect species (Wernegreen, 2012; Sinclair et al., 2015; Alford et al., 2018). Climate change models also predict increases in freeze-thaw cycles corresponding to reduced snow cover, which would have a direct impact on the microenvironment of many soil-dwelling insects (Sinclair, 2001). Thus, studies examining temperature tolerance and the mechanisms that contribute to this tolerance are becoming increasingly important, especially if we are to estimate how insect populations might respond in the future. As poikilotherms, insects are particularly susceptible to fluctuations in temperature which has led to the evolution of various thermal tolerance strategies to maintain internal homeostasis and preserve metabolic functions (Kokou et al., 2018). The level of vulnerability to fluctuations in environmental conditions (e.g., increased exposure to thermal stress) for various insect species has been linked with the underlying genetic and phenotypic plasticity present in these organisms, which play a direct role in mediating host physiological response (Macke et al., 2017; Kokou et al., 2018).

The diversity of mechanisms used by insects to acclimate to novel environments or shifts in environmental conditions, include examples of behavioral modifications (e.g., habitat selection, temporal activity), physiological changes (e.g., production of enzymes, antioxidants, proteins, lipids, carbohydrates, trehalose, etc.), and/or phenotypic alterations (e.g., reproduction, diapause, seasonal phenology) (Kingsolver and Huey, 1998; Huey et al., 2003; Danks, 2005; Clark and Worland, 2008; Huey, 2010; Lacey et al., 2010). There is also growing evidence linking insect-associated microorganisms with thermal tolerance, specifically the contributions of gut microbiota as members of this community are closely tied to overall host fitness (Ferguson et al., 2018; Zhang et al., 2019; Sepulveda and Moeller, 2020; Jaramillo and Castañeda, 2021). Some of the early studies on the role of microorganisms in cold tolerance focused on interannual shifts associated with phenology. For example, in certain freeze-tolerant insects, the presence of ice nucleating bacteria was found to regulate freezing in preparation for overwintering (Strong-Gunderson et al., 1990; Lee et al., 1991, 1993; Wernegreen, 2012; Moghadam et al., 2018). Conversely, in non-freeze-tolerant insects, these ice nucleating bacteria may be excreted in preparation for cold temperatures to prevent freezing (Lee et al., 1996). Gut microbiota can also indirectly impact thermal tolerance by shifting the relative abundance of certain microbial lineages to those that facilitate a physiological response that promotes tolerance to thermal stress (Sepulveda and Moeller, 2020). Ferguson et al. (2018), for example, found seasonal shifts in gut microbiota in crickets (*Gryllus veletis* Alexander and Bigelow) to be linked to increased freeze tolerance and immunity. Raza et al. (2020) showed gut microbiota in tephritid fruit flies [*Bactrocera dorsalis* (Hendel)] contributed to resistance to low temperature stress by stimulating the arginine and proline metabolism pathway in the insect host, leading to elevated levels of certain cryoprotectant transcripts and metabolites.

However, while associations with certain microbial taxa can foster phenotypic plasticity that increases tolerance to thermal stress, maintaining these microbial associations has the potential to be evolutionarily costly. Alterations to the gut microbial community could modify the functional relationships between gut microbiota and the insect host, leading to a shift in their contribution to other host functions and physiological responses (Sepulveda and Moeller, 2020). This can have a deleterious impact on host fitness if the result is selection against microbial symbionts involved in metabolism, nutrient acquisition, resistance to pathogens, host growth/development, or reproduction (Sepulveda and Moeller, 2020). In fact, it has been suggested that primary symbionts of certain insect species (i.e., microorganisms that perform specific, essential functions that influence host fitness), can actually constrain, rather than facilitate, thermal tolerance (Wernegreen, 2012; Zhang et al., 2019). Wernegreen (2012) hypothesized that because symbionts have co-evolved with their host, they are physiologically and genetically constrained, and thus more susceptible to environmental fluctuations. Some researchers have demonstrated a negative effect of temperature exposure on primary bacterial symbionts. Prado et al. (2010) found a significant reduction in bacterial symbionts in the guts of two stinkbug species (*Acrosternum hilare* and *Murgantia histrionica*) after high temperature exposure (30°C). Montllor et al. (2002) found guts of heat-stressed pea aphids to have a reduced number of bacteriocytes which house their obligate primary symbiont, *Buchnera*. However, the degree to which these symbionts act to constrain host thermal tolerance is still very much unknown. It is likely that microbially- mediated thermal tolerance can occur when there is enough flexibility in microbial diversity to respond to shifting environmental conditions, while also maintaining the core microbial community required for host functioning (Macke et al., 2017).

Perhaps one of the most studied insect-symbiont relationships is that of wood-feeding termites and their gut symbionts, which are essential for breakdown of lignocellulosic materials (Breznak and Brune, 1994; Radek, 1999; Brune, 2014). In North America, subterranean termites in the family Rhinotermitidae are responsible for the majority of economic costs associated with damage to wooden products and structures (Su and Scheffrahn, 2000). Members of Rhinotermitidae are broadly distributed in both temperate and tropical regions, although individual termite species and populations tend to thrive within relatively narrow temperature ranges and under specific environmental conditions (Esenther, 1969; Davis and Kamble, 1994). As microbial symbionts have been credited as significant drivers of species evolution and diversification in other social insects (Mueller et al., 2011), there is the potential for termite-associated microorganisms to influence thermal tolerance plasticity and constrain or expand termite geographical distributions.

A number of researchers dating back to the 1920s have examined the effect of temperature on termites (Esenther, 1969; Haverty and Nutting, 1974; Sponsler and Appel, 1991; Davis and Kamble, 1994; Cabrera and Kamble, 2001; Hu and Appel, 2004; Hu and Song, 2007; Gautam and Henderson, 2011), as well as on their symbiotic gut protozoa (Cleveland, 1923, 1924; Cook and Smith, 1942; Mannesmann, 1969; Smythe and Williams, 1972; Belitz and Waller, 1998; Cabrera and Kamble, 2004). Cleveland (1924) showed that all gut protozoa were killed after 24-h incubation at 36°C in *Reticulitermes flavipes* (Kollar). Smythe and Williams (1972) observed no effect on protozoa at 29°C and report the highest tolerable temperature range for *R. flavipes* survival to be between 31.5 and 33°C. Despite this abundance of research relating to the effect of temperature on protozoa, the effect on gut bacteria has been mostly ignored. One exception to this is a study of termite cold tolerance, where Cabrera and Kamble (2004) found the supercooling point (SCP) to be the lowest in *R. flavipes* workers treated with antibiotics, and hypothesized that symbionts (either protists or bacteria) may be acting as ice nucleators in the termite hindgut.

In this work we examine the effect of temperature acclimation on shifts in the microbial community of subterranean termite guts as well as in termite-manipulated nest/soil. Soil material was included in this work as it is the physical substrate used by termites in construction of tunnels and galleries in the nest. It is comprised of termite feces and salivary secretions which bind the nest together and thus, can potentially provide insight into the fraction of the termite gut microbiome that might be evacuated under certain temperature conditions. The primary objectives of this study were to: (1) examine the physiological performance of eastern subterranean termites, *R. flavipes*, after prolonged exposure to three different temperature conditions using measurements of feeding activity, mortality, and cold tolerance [SCP and critical thermal minimum (CTmin)], (2) evaluate how an increase or decrease in temperature affects the microbial community of the termite gut and associated nest material, and (3) examine how these microbial shifts relate to cold tolerance. These objectives aim to gain a better understanding of temperature induced physiological and microbiological shifts of the termite gut microbial community and their potential functional role in termite thermal tolerance.

## Materials and Methods

### Experimental Set-Up and Temperature Treatment

Termite experiments were performed in sterile plastic Petri dishes containing sterile soil (10 g), and sterile DI water (1 mL). A southern yellow pine wood block (*Pinus* spp. mix – *P. echinata* Mill., *P. elliottii* Engelm., *P. taeda* L.) (40 mm × 25 mm × 2 mm) which was conditioned at 27°C/30% relative humidity (RH), weighed and autoclave-sterilized, was included in each dish as a food source. A total of 100 *R. flavipes* workers from a laboratory colony were added to each dish which was then sealed with parafilm to maintain moisture levels (test start March 2018). These laboratory termites were comprised of a compilation of individuals collected in corrugated cardboard traps from Janesville, Wisconsin between the months of May and November 2017. Termites were maintained under constant conditions in an environmental chamber (27°C, 80% RH) prior to use. Termites were exposed for 4-weeks to one of three temperatures characterized as low (15°C), medium (27°C), or high (35°C), with five replicate dishes per temperature treatment. These temperatures were selected based on experimental ranges used by previous researchers that elicited physiological effects in laboratory termite tests.

### Termite Guts and Soil Samples

After 4-weeks, soil material (0.25 g) manipulated by the termites (i.e., soil pasted to the side of the container by termites using salivary secretions and feces) was sterilely collected in duplicate from each of the petri dishes into sterile 2 mL microcentrifuge tubes. Pine wood feeder blocks from each dish were brushed free of debris, re-conditioned to a constant weight and re-weighed to determine the amount of termite feeding at each temperature treatment. Termites from each dish were counted to determine mortality. Individual termites were then randomly selected from each of the five replicate containers for each temperature group for use in cold tolerance assays (*n* = 31 termites per treatment group). Another set of individual termites were randomly selected for 16S rRNA amplicon sequencing. These samples consisted of 20 termites from each of the five replicate containers for all treatment groups, with the exception of one of the five test replicates belonging to the high temperature set as all termites were dead at the end of the test period. Preparation of these samples for sequencing involved first sacrificing termites by freezing (∼10 min. at 0°C) followed by a surface rinse in 70% EtOH. Gut dissections were done by pinching the tip of the last abdominal segment and gently pulling until the guts separated from the rest of the abdomen. Guts were pooled so that each sample consisted of 20 termite guts per replicate container (i.e., five tubes of 20 guts per temperature group, except for high temperature samples which had four tubes of 20 guts because of termite mortality). In addition to these experimental samples, extracted guts were pooled from 20 termite workers collected directly from a field site in Janesville, Wisconsin at 11 time points across late spring, summer and early winter seasons for comparison to lab termites used in the temperature exposure tests.

### DNA Extraction and 16S rRNA Amplicon Sequencing

Termite guts were extracted directly into ZR bashing bead lysis tubes (Zymo Research, Irvine, CA, United States) and a mix of 600 μl of tissue and cell lysis solution and 2 μl Proteinase K from the MasterPure Complete DNA and RNA Purification Kit (Epicenter-Lucigen, Middleton, WI, United States) was added to each sample tube. All tubes were then vortexed vigorously for 2 min. Hereafter, DNA extraction methods followed the Epicenter-Lucigen total nucleic acids purification protocol. DNA was extracted from 0.25 g of collected soil material using the PowerSoil DNA Isolation Kit (MoBio, Carlsbad, CA, United States) following the provided protocol. Both termite gut and soil DNA was quantified using the Qubit Fluorometer (HS-assay kit; Invitrogen, Carlsbad, CA, United States) and diluted to 20 ng/μl.

Polymerase chain reaction (PCR) was done using tagged MiSeq primers targeting the V4 region of the 16S rRNA gene (primers: forward – GTGCCAGCMGCCGCGGTAA; reverse – GGACTACHVGGGTWTCTAAT) (Kozich et al., 2013). Reaction mixtures for each sample (24 μl) included 12 μl KAPA HiFi HotStart ReadyMix (2X) (KAPA Biosystems, Boston, MA, United States), 1.5 μl NanoPure water, 1 μl forward primer (10 μM), 1 μl reverse primer (10 μM), and 8 μl diluted DNA (20 ng/μl). All samples were run in triplicate under the following conditions: initial denaturation at 95°C for 3 min, 25 cycles of 95°C for 30 s, 55°C for 30 s, 72°C for 30 s, and a final elongation step at 72°C for 5 min. A non-template control reaction was included and submitted for sequencing.

Amplicon libraries were sequenced with the paired-end Illumina MiSeq platform at the University of Wisconsin-Madison Biotechnology Center. Reads were preprocessed, assembled, aligned, and classified using the mothur pipeline (Schloss et al., 2009). Classification was based on the Silva SEED release 132 database. Operational taxonomic units (OTUs) were defined using a 99% similarity threshold. A heatmap was built using *Z*-score normalized data was calculated as *Z* = (x−u)/d, where *x* is the relative abundance of a taxa, *u* is the mean relative abundance of a taxa across all the samples and, *d* is the standard deviation across all the samples. A phylogenetic tree of representative sequences (250 bp) of the top OTUs across all samples was performed in the MEGA v7 using the maximum likelihood method with 500 bootstrap replicates, assuming a General Time Reversible (GTR) substitution model. Using the OTU table with data not normalized or rarefied, OTU richness was estimated using Chao-1 index (Chao and Chiu, 2016), and non-metric multidimensional scaling (nMDS) and permutational analysis of variance (PERMANOVA) were used to quantify differences among samples and assess statistical significance, using PRIMER-e v 7.0.13. Differentially abundant OTUs were identified using MaAsLin2 (Microbiome Multivariable Associations with Linear Models) (Mallick et al., 2021).

### Thermal Tolerance Tests

Termites used for this study originated from a population of *R. flavipes* along the northern range of its distribution where they are likely limited by cold seasonal conditions. Thus, two cold tolerance assays, SCP and CTmin, were selected as the physiological measures of thermal tolerance.

#### Supercooling Point

Two worker termites (3rd or 4th instar) were placed into each of eight 1.5 ml microcentrifuge tubes (*n* = 16 per treatment group). *T*-type thermocouples were wrapped near the base with cotton rounds and pushed in, next to the termites at the bottom of the tube to measure termite temperature. The other end of the thermocouple was inserted into an 8-channel TC-08 Data Logger (Pico Technology, Tyler, TX, United States) set to record temperature of all eight channels, at a sampling interval of 2 s. Tubes were then inserted into a floating tube rack and carefully placed into the refrigerated water bath (Grant Instruments, Cambridge, United Kingdom) containing 50:50 propylene glycol to water. The water bath was controlled using an external circulating pump and accompanying LabWise software, which was programmed for an initial 10-min acclimation step at 20°C followed by a decrease in temperature at a rate of 0.5°C/min down to 5°C, then at 0.2°C/min until it reached −15°C. Measurements of SCP were determined by a spike in temperature as measured by the data logger, representing the temperature at which freezing was initiated.

#### Critical Thermal Minimum

A custom aluminum cooling block was connected to a refrigerated water bath (Grant Instruments, Cambridge, United Kingdom) with insulated plastic tubes, allowing for cycling of the 50:50 propylene glycol to water between the water bath and aluminum block. Water bath temperature was controlled using an external circulating pump and accompanying LabWise software, programmed for an initial 10-min acclimation step at 20°C followed by decreasing temperatures at a rate of 0.2°C/min down to −15°C. Groups of three termite workers (3rd or 4th instar) were added to five of the six small culture plates attached to the center of the aluminum cooling block with thermal conducting tape (*n* = 15 per treatment group). Each culture plate had a small hole drilled in the lid to prevent condensation and so that a *T*-type thermocouple could be inserted for accurate temperature measurements. Temperature was recorded using a TC-08 Data Logger (Pico Technology, Tyler, TX, United States) in one of the six culture plates. Measurements of CTmin were determined as the temperature at which termites displayed a lack of righting ability after being gently flipped over with a paint brush, indicating that they could no longer coordinate their muscle movements. Once CTmin was reached, the termite was removed from the test dish into a 12-well plate and allowed to recover at room temperature (note: if termites did not recover, the CTmin threshold was passed and the data from that termite was deemed invalid).

## Results and Discussion

### Effect on the Termite

#### Survival and Feeding

**FIGURE 1:**
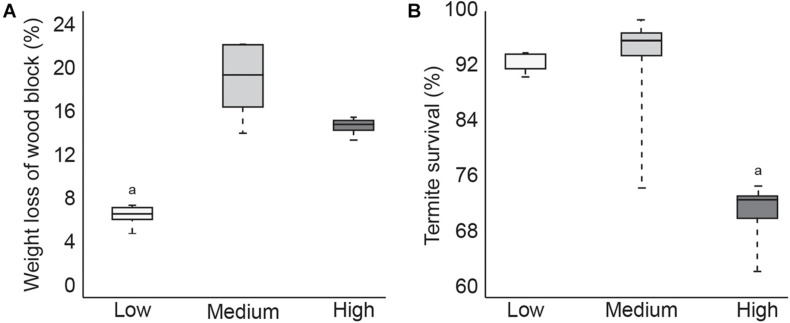
Boxplot showing average1pc amount of feeding **(A)** and average termite survival **(B)** after 4 weeks at low, medium, or high temperatures (*p* < 0.05).

During the 4-week test, termites in the low temperature group consumed significantly less of the pine wood feeder block compared to termites in the other two temperature treatment groups (Figure 1A). Termite survival was comparable between the low and medium temperature groups but was significantly lower in termites exposed to the high temperature treatment, with 100% mortality in one of the five test containers (Figure 1B). These results suggest that while termites ate less at lower temperatures, this did not negatively impact survival. Termites in the high temperature group consumed slightly less wood material than the medium temperature termites, which was probably the result of increased mortality. Since wood consumption was not significantly lower however, it is likely that termite mortality occurred toward the end of the test period.

#### Cold Tolerance

**FIGURE 2:**
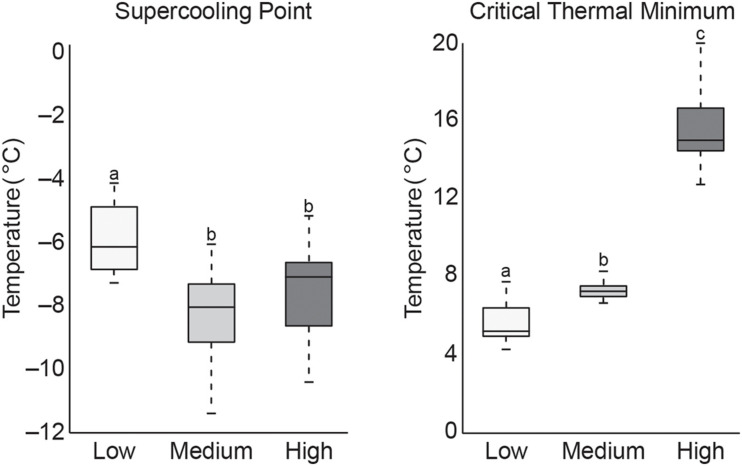
Boxplot of SCP and CTmin values of termite workers after 4 weeks of exposure to low, medium, or high temperatures (treatments that do not share the same letter are significantly different *p* ≤ 0.05).

Data from cold tolerance tests, SCP and CTmin are shown in Figure 2. These tests showed a significantly higher CTmin (mean 15.6°C) in high temperature exposed termites, indicating increased susceptibility to colder temperature. Termites acclimated at low temperature had a significantly lower CTmin (mean 5.7°C), but a significant increase in SCP (mean −5.9°C) compared to termites acclimated at the medium (mean CTmin 7.3°C/SCP −8.4°C) or high (mean SCP −7.6°C) temperatures. This suggests that pre-exposure to cold allowed termites to stay active longer in decreasing temperatures but caused termites to freeze more easily. These CTmin results might be indicative of cold tolerance adaptability in these northern termite populations by allowing for foraging and nest maintenance activities to continue into the colder, winter months. However, additional research is needed to confirm this hypothesis.

The higher SCP values recorded from the low temperature group suggest an increase in freezing susceptibility. However, this is not altogether unexpected as termites are thought to be freeze-intolerant/freeze avoidant insects (Mail, 1930; Esenther, 1969; Cabrera and Kamble, 2001, 2004; Hu and Song, 2007; Clarke et al., 2013) and SCP represents a lower limit of survival that may be dependent on multiple physiological factors (Renault et al., 2002; Lee, 2010). Results from this study are supported by those from a similar study of *R. flavipes* cold tolerance where they showed an increase in SCP after pre-exposure to 10°C, but concluded that lowering SCP is not likely a factor in cold acclimation as termites are not freeze tolerant insects (Davis and Kamble, 1994). While our results reinforce this conclusion, the reason for higher SCP values after cold acclimation remains unknown but may relate to the abundance of ice-nucleating agents in the termite gut. Other freeze-intolerant insect species have been shown to reduce ice nucleating bacteria and/or microbially produced compounds (e.g., calcium carbonate, potassium phosphate, uric acid, certain amino acids, proteins, steroids) which lead to freezing injury (Vonnegut, 1949; Head, 1961; Strong-Gunderson et al., 1990; Kawahara, 2002; Clark and Worland, 2008; Lee, 2010). When ice nucleators are present, they promote freezing at higher temperatures, which serves to limit ice formation to extracellular fluids in freeze tolerant species (Neven et al., 1989; Duman et al., 2010). Thus, high SCP in low temperature groups might be indicative of the presence of ice nucleating microorganisms that either increased during the cold acclimation period or are normally excreted seasonally in field termites in preparation for winter. Accordingly, lower SCP values in the other two temperature groups may be the result of reduction or shift in gut microbiota that would otherwise act as ice nucleating agents. Cabrera and Kamble (2004) showed antibiotic-treated *R. flavipes* workers to have the lowest SCP values and suggest this may be the result of removing ice-nucleating microorganisms. The significance of these results remains to be determined but could hint at one possible transition by which freeze intolerant insects, such as termites, may evolve to become freeze tolerant. Studies of field populations that examine seasonal shifts in gut microbiota and associated nest material across their geographical range are needed to better understand how termite-microbe interactions might be contributing to thermal tolerance.

### Effect on the Termite Gut

#### Similarity and Diversity: Gut Microbial Community

**FIGURE 3:**
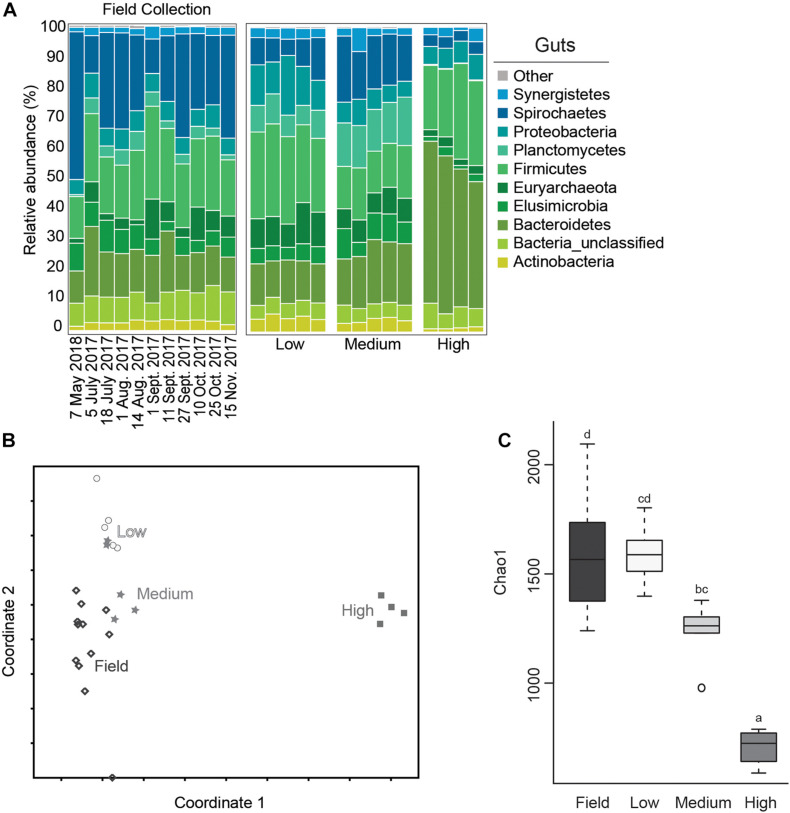
Average relative abundance (%) of bacterial phyla from field collected termite guts and after temperature exposure **(A)**; Non-metric Multidimensional Scaling (NMDS) plot based on the Bray-Curtis distance of the bacterial communities of termite guts after temperature treatment and from field collected samples **(B)**; boxplot showing estimated microbial diversity based on Chao-1 for guts from field collected samples and those exposed to temperature treatments **(C)** (treatments that do not share the same letter are significantly different *p* ≤ 0.05).

Relative abundance of bacterial phyla from each of the test groups and from field collected gut samples are shown in Figure 3. The microbial community of field collected *R. flavipes* guts were dominated by Spirochaetes, followed by Firmicutes, Bacteroidetes, Elusimicrobia, and Proteobacteria, which is comparable to results from other studies that characterize the core microbiota of *Reticulitermes* spp. guts (Ohkuma and Brune, 2011; Boucias et al., 2013; Brune, 2014; Benjamino and Graf, 2016). This community seems to stay relatively constant across sampling dates. Within the temperature treatment groups, the microbial community of guts from the medium temperature groups most closely resembled those of field populations at the phyla level although they did show a small reduction in the relative abundance of Spirochaetes and an increase in Planctomycetes (Figures 3A,B). This is important as the medium temperature value selected for this study is the same as the temperature setting used for maintaining termite populations in the lab. This suggests that there is some shift in gut microbiota in laboratory termite colonies compared to those directly from the field, but not nearly to the degree observed with low or high temperature treatments.

**FIGURE 4:**
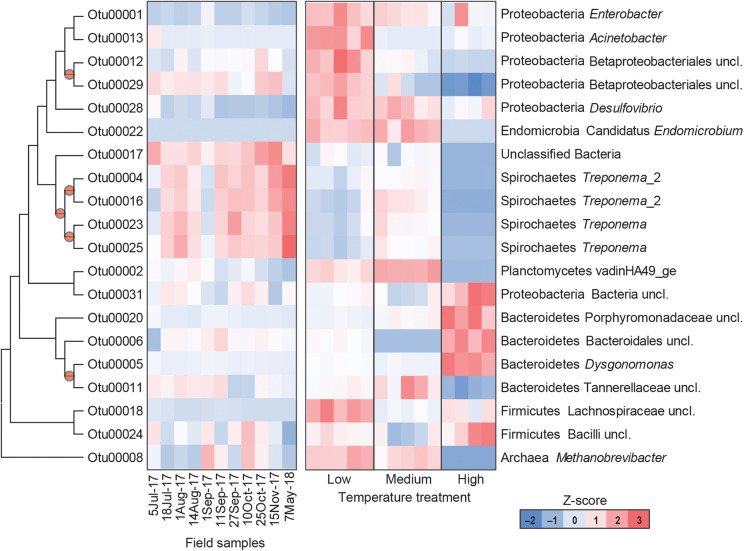
Heatmap representing the relative abundance of the top 20 OTUs from field collected termite guts (left) and termite guts after low, medium, or high temperature exposure (right). Columns represent different samples, and rows represent *Z*-score normalized abundance of OTUs and are clustered based on a maximum likelihood phylogenetic tree of OTU representative sequences. All OTUs shown in this figure are differentially abundant among treatments [MaAsLin, *p* < 0.001, maximum false discovery rate (significance threshold) < 0.05]. Nodes with bootstrap values > 0.7 are marked with a circle.

The gut microbial community composition was significantly more similar within treatments than between treatment groups (Tables 1, 2 and Figure 3). However, termite guts from the medium and low temperature groups showed more similarity to each other, and to those collected directly from the field, than termite guts from the high temperature group. This suggests that the high temperature treatment caused the most dramatic shift in the overall microbial community of the termite gut, specifically an increased relative abundance of Bacteroidetes to nearly 50% of the gut community composition (Table 1 and Figure 3A). For the distribution of all OTUs across samples see Supplementary Table 1. Examination of the top 20 OTUs from gut samples of all temperature treatment groups, showed four OTUs to be members of Bacteroidetes, three of which were increased in high temperature exposed termites (Figure 4). Two belong to Dysgonomonadaceae (OTUs: 00020, 00005), a family that has recently been shown to contain ectosymbionts of intestinal nematodes in wood-feeding cockroaches (Murakami et al., 2019), although their significance in this study remains to be determined. Additionally, Chao-1 richness was significantly reduced in guts from the high temperature group compared to those from field samples or from the other two temperature groups, suggesting decreased species diversity overall (Figure 3C). Other studies have also shown the gut of poikilotherms to have lower bacterial diversity after exposure to heat stress (Bestion et al., 2017; Fontaine et al., 2018). Studies in bivalves (Abele et al., 2002) and lepidopterans (Cui et al., 2011) have demonstrated that exposure to thermal stress can lead to an increase in the production of reactive oxygen species. Therefore, one possible explanation for the observed lower bacterial diversity under high temperatures is that different species of gut symbionts show different levels of tolerance to these types of intrinsic factors produced by the host (e.g., reactive oxygen species, hormones, heat-shock proteins), all of which could result in changes in microbiome structure (Obata et al., 2018).

**TABLE 1:** Average Bray-Curtis similarity (%) of the bacterial communities within and between groups.

Average similarity	Treatment	Soil	Guts
Within	Field	–	58.4
	Low	42.9	63.7
	Medium	52.2	64.7
	High	49.0	64.6
Between	Field-Low	–	50.2
	Field-Medium	–	50.0
	Field-High	–	30.4
	Low-Medium	27.5	57.2
	Low-High	25.0	34.7
	Medium-High	46.7	40.0

**TABLE 2:** Pair-wise PERMANOVA results for Bray-Curtis similarities of the bacterial communities from the termite guts and termite-manipulated soil materials after temperature treatment (low, medium, or high) and from field collected samples.

	Comparison groups	*t*	*P*-value
Termite guts	Field, High	3.5506	0.001
	Field, Low	2.1921	0.002
	Field, Medium	2.2398	0.002
	High, Low	3.3492	0.01
	High, Medium	3.0541	0.006
	Low, Medium	1.7686	0.008
Soil	High, Low	2.2329	0.009
	High, Medium	1.3191	0.006
	Low, Medium	2.3433	0.008

Results from this study also suggest a potential role of thermal stress in shaping the prokaryotic community associated with termite–protist symbionts. Phylogenetically lower termites maintain a diversity of gut protists that are essential for breakdown of lignocellulosic materials in wood-feeding termites, and thus essential for termite survival (Breznak and Brune, 1994; Hongoh, 2011; Brune, 2014). Here, high temperature treated groups showed reduced relative abundance of phyla that contain a number of protist-associated species, including Spirochaetes, Elusimicrobia, and Euryarchaeota. Within the Spirochaetes, species of *Treponema* represent members of the core microbial community in guts of *Reticulitermes* spp. where they are thought to either serve as CO_2_ reducing acetogens or contribute to fixation of atmospheric nitrogen (N_2_) (Noda et al., 2003; Benjamino and Graf, 2016). Examination of the 20 most abundant OTUs from all groups, four were identified as *Treponema* spp. (OTUs: 00004, 00016, 00023, and 00025) all of which were reduced in the high temperature treatment group (Figure 4). Decreased relative abundance of specific OTUs after high temperature exposure was also observed in Elusimicrobia candidate genus *Endomicrobium* (OTU00022) which are known cytoplasmic protist endosymbionts (Stingl et al., 2005) and in a phylum of methanogenic archaea, Euryarchaeota *Methanobrevibacter* (OTU00008). Members of Euryarchaeota have been identified from the guts of all extant families of termites (Purdy, 2007), and have mostly been found as ecto- and endosymbionts of termite gut protozoa although some have been shown to be associated with the gut wall (Ohkuma et al., 2006). Species of *Methanobrevibacter* specifically have been repeatedly identified from termite guts where they are suggested to utilize H_2_ and CO_2_ (Tokura et al., 2000). Thus, these data suggest that high temperatures likely have a negative impact termite gut protozoa, which agrees with results from Smythe and Williams (1972) who showed reductions in symbiotic protists (particularly those in the genera *Pyrsonympha* and *Dinenympha*) in guts of subterranean termites held at temperatures between 30 and 31.5°C. One possible explanation for this is that heat stress induced production of reactive oxygen species would negatively affect flagellate gut protists that require a strict anaerobic environment for survival. As the external and internal surfaces of these flagellate protists have been shown to serve as attachment sites for numerous gut bacteria, a loss of protozoa would result in a loss of habitat for these bacterial species. Overall, the relative abundance of 802 OTUs were found to be significantly different in the termite guts from the different treatments (Supplementary Table 2). These results support the hypothesis that the deleterious effects from high temperature exposure on termite survival may be indirectly related to the thermosensitivity of the microbial symbionts of the termite host.

Guts from low temperature treatment groups showed a reduction in the relative abundance of Spirochaetes and an increase in the relative abundance of Firmicutes, Lachnospiraceae (OTU 00018), in particular. The reduction in Spirochaetes could be related to decreased feeding at low temperature suggesting that less energy is being utilized for nutrient acquisition perhaps in favor of microbial associations linked to an increase in cold-tolerance. Low temperature gut samples also showed a dramatic shift in Proteobacteria, having nearly twice the relative abundance of Proteobacteria compared to the other temperature groups or field samples, specifically *Acinetobacter* (OTU00013), *Desulfovibrio* (OTU00028), Betaproteobacteriales (OTU00012, OTU00029), and *Enterobacter* (OUT00001) (Figure 4). These data agree with a study examining seasonal shifts in the gut microbiome of spring field crickets that also showed an increase in the relative abundance of Proteobacteria after cold temperature exposure (Ferguson et al., 2018). This association of Proteobacteria and cold temperature exposure may correspond to the known ice nucleating activity of certain members of this phylum (e.g., *Pseudomonas* spp., *Enterobacter* spp., *Xanthomonas* spp.) (Lee et al., 1991, 1993, 1995). Therefore, the increased relative abundance of Proteobacteria in termite guts from the low temperature group may relate to cold tolerance, specifically the increase in SCP in termites pre-exposed to cold.

### Effect on Termite-Manipulated Soil

#### Similarity and Diversity: Soil Microbial Community

**FIGURE 5:**
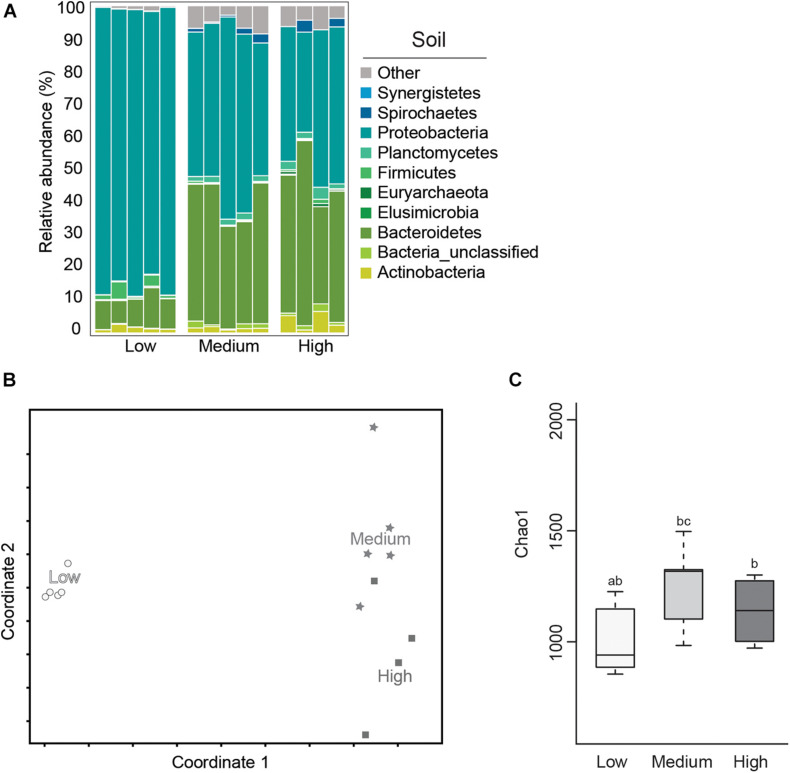
Average relative abundance (%) of bacterial phyla **(A)**; Non-metric Multidimensional Scaling (NMDS) plot based on the Bray–Curtis distance of the bacterial communities **(B)**; and boxplot showing estimated microbial diversity based on Chao-1 **(C)**, for termite-manipulated soil materials after temperature treatment (treatments that do not share the same letter are significantly different *p* ≤ 0.05).

Microbial diversity and average relative abundance of bacterial phyla from termite-manipulated soil material are shown in Figure 5. It is important to note that as soil materials used in this study were autoclaved prior to testing, the soil microbial community presented here is more representative of organisms associated with the termite cuticle or shed from the gut during tunneling/nest building activity than the microbial community composition under natural conditions. Further experiments using non-sterile soil, as well as field studies, would be needed to address how the soil microbial community shifts in the termite nest under natural conditions where they would be subjected to more gradual changes in environmental conditions as well as competition from other soil microorganisms. Therefore, results from this work should be considered, at least in part, in terms of how they compare to data from termite gut samples under the different temperature conditions.

In this study, soil samples from the low temperature group showed lower microbial diversity compared to the other temperature treatments based on Chao-1 (Figure 5 and Table 1). Comparisons of soil samples showed a clear shift in the microbial community after low temperature treatment compared to the distinct, but more similar communities from the high and medium temperature samples (Figures 5A,B and Tables 1, 2). A predominance of Proteobacteria and Bacteroidetes occurred in all soil samples, although the latter was reduced in soil from the low temperature group. As observed in termite gut samples, the relative abundance of Proteobacteria was also highest in soil samples from the low temperature groups. Soil material from the high temperature group had an increased abundance of Planctomyctes and Actinobacteria, which is contrary to the decreased abundance of these phyla observed in the corresponding termite gut samples.

In total, the relative abundance of 181 OTUs were found to be significantly different in the soil samples of the different treatments (Supplementary Table 2). Among the OTUs associated with the high temperature soil samples, were OTU00313, Actinobacteria – *Kitasatospora*, and OTU01388, an unclassified member of Firmicutes, order Clostridiales. Many members of both Actinobacteria and Firmicutes are able to form spores that can survive autoclaving (Hirvelä-Koski, 2004; Otte et al., 2018). Therefore, we hypothesize that spores of these bacteria may have been present in the soil before autoclaving and that high temperature treatment was favorable for spore germination. This is corroborated by the fact that these OTUs were virtually absent in termite gut samples (Supplementary Table 2). Two Proteobacteria [*Enterobacter* (OTU00001) and *Acinetobacter* (OTU00013)] dominated the low temperature soil samples and were also associated with low temperature guts. These results are in alignment with a number of recently published studies that have noted shifts in the relative abundance of bacterial taxa belonging to Proteobacteria associated with thermal stress in a variety of host species (Sepulveda and Moeller, 2020). Further studies are needed however, to determine the relationship between members of Proteobacteria and cold tolerance in subterranean termites.

### Implications for Cold-Tolerance in Northern Termite Colonies

It has been suggested that northern populations of subterranean termites exhibit differences in reproductive biology, colony formation, and dispersal compared to their southern counterparts (Esenther, 1969). However, the various factors that contribute to these differences remain unknown. Many northern termite colonies are thought to have been introduced initially by way of infested wood materials, with subsequent colony growth occurring primarily through secondary reproductives rather than winged alates (Esenther, 1969; Raffoul et al., 2011). Initially, this led to assumptions that these populations would have lower genetic diversity caused by geographical isolation and from a lack of immigration or emigration. Previous work, however, showed Wisconsin termite populations to have higher amounts of within colony genetic variation than expected, suggesting that intraspecific genetic variation is likely to be a major factor in successful colony establishment in northern climates (Arango et al., 2015). It has been hypothesized that there is a threshold relating to genetic diversity, below which termite colonies are not able to persist (DeHeer and Vargo, 2006). Further studies are needed to examine the possible effect of host genotype on associated gut microbial diversity in relation to thermal tolerance. This information could lead to a better understanding how termite colonies become established along their northern range and the factors that subsequently support colony persistence and growth. For example, cold winter temperatures is often identified as a potential factor effecting termite dispersal by promoting caste differentiation into secondary reproductives over winged alates (Esenther, 1969; Arango et al., 2015). As gut microbiota have been shown to foster phenotypic plasticity (Macke et al., 2017), it is possible that this observed variation in termite reproductive biology may be linked with, or facilitated by, members of the gut microbial community. Studies examining gut microbial diversity in combination with cold tolerance physiology between termite castes may provide some insight into this phenomenon.

## Conclusion

In this study, we found shifts in environmental temperature can cause substantial changes in the microbial community of *R. flavipes* guts as well as in associated soil/nest materials, particularly among members of Proteobacteria, Bacteroidetes, and Firmicutes. Our results suggest that northern termite populations might be more vulnerable to high heat exposure through microbial community loss/shifts, compared to low temperature. Thus, it is conceivable that *R. flavipes* activity might decline in warmer, southern regions (assuming similarity in gut microbial structure), and northern range edges might expand. However, the opposite may be true if southern termite populations were to be tested in a similar manner. Using an acclimation based approach, we were not yet able to establish a cause-effect relationship between these findings, and suggest that more studies are needed to investigate how the termite gut microbiota can modulate the host’s physiological acclimation to temperature changes and vice-versa. Future work should include field studies examining shifts in gut-microbe communities from termite populations across seasonal and geographical gradients as they may lead to a better understanding of the potential contribution of gut microbiota in adaptive phenotypic plasticity under more ecologically relevant conditions. Additionally, research is needed to determine how changes to gut microbiota might impact overall host fitness by evaluating shifts in microbial community functionality. Specifically, how disruptions to the termite gut microbiome effects other essential processes (e.g., metabolism/nutrient acquisition, resistance to pathogens, growth/development, and reproduction).

## Data Availability Statement

The datasets presented in this study can be found in online repositories. The name of the repository and accession number can be found below: National Center for Biotechnology Information (NCBI) BioProject, https://www.ncbi.nlm.nih.gov/bioproject/, PRJNA680366.

## Author Contributions

RA, SS, CC, and CC-S: conceptualization, methodology, and draft revision and editing. RA and CC-S: investigation, analysis, and writing and draft preparation. All authors have approved the publication of this manuscript.

## Conflict of Interest

The authors declare that the research was conducted in the absence of any commercial or financial relationships that could be construed as a potential conflict of interest.

## Supplementary Material

Supplementary Table 1 Distribution of OTUs for all samples across treatment and sample types.

Supplementary Table 2 Differentially abundant OTUs based on MaAsLin2 analysis.
